# Ethyl 3,6-dihy­droxy-6-methyl-4-phenyl-4,5,6,7-tetra­hydro-1*H*-indazole-5-carboxyl­ate monohydrate

**DOI:** 10.1107/S160053681100242X

**Published:** 2011-01-22

**Authors:** Abel M. Maharramov, Arif I. Ismiyev, Bahruz A. Rashidov

**Affiliations:** a Baku State University, Z. Khalilov St. 23, Baku AZ-1148, Azerbaijan

## Abstract

In the title compound, C_17_H_20_N_2_O_4_·H_2_O, the cyclo­hexene ring adopts a half-chair conformation while the indazole ring is essentially planar [maximum deviation = 0.0192 (12) Å]. In the crystal, pairs of inter­molecular O—H⋯N hydrogen bonds link the mol­ecules into dimers lying about inversion centers and intra­molecular O—H⋯O hydrogen bonds result in six-membered rings. The dimers are further connected by N—H⋯O and O—H⋯O hydrogen bonds.

## Related literature

For general background to azoles, see: Genin *et al.* (2000 ▶). For a related structure, see: Hema *et al.* (2006 ▶).
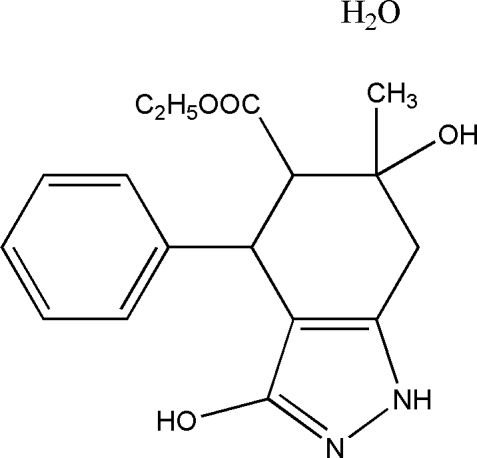


## Experimental

### 

#### Crystal data

C_17_H_20_N_2_O_4_·H_2_O
*M* *_r_* = 334.37
Triclinic, 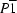

*a* = 6.9964 (15) Å
*b* = 8.8647 (19) Å
*c* = 15.124 (4) Å
α = 99.363 (6)°
β = 95.281 (6)°
γ = 112.271 (4)°
*V* = 844.2 (3) Å^3^
*Z* = 2
Mo *K*α radiation
μ = 0.10 mm^−1^
*T* = 296 K
0.20 × 0.20 × 0.20 mm

#### Data collection

Bruker APEXII CCD diffractometer
Absorption correction: multi-scan (*SADABS*; Bruker, 2003 ▶) *T*_min_ = 0.981, *T*_max_ = 0.981
6332 measured reflections
2889 independent reflections
2327 reflections with *I* > 2σ(*I*)
*R*_int_ = 0.065

#### Refinement

*R*[*F* ^2^ > 2σ(*F* ^2^)] = 0.077
*wR*(*F* ^2^) = 0.198
*S* = 1.00
2889 reflections
231 parameters
H atoms treated by a mixture of independent and constrained refinement
Δρ_max_ = 0.27 e Å^−3^
Δρ_min_ = −0.37 e Å^−3^

### 

Data collection: *APEX2* (Bruker, 2005 ▶); cell refinement: *SAINT-Plus* (Bruker, 2001 ▶); data reduction: *SAINT-Plus*; program(s) used to solve structure: *SHELXS97* (Sheldrick, 2008 ▶); program(s) used to refine structure: *SHELXL97* (Sheldrick, 2008 ▶); molecular graphics: *SHELXTL* (Sheldrick, 2008 ▶); software used to prepare material for publication: *SHELXTL*.

## Supplementary Material

Crystal structure: contains datablocks global, I. DOI: 10.1107/S160053681100242X/pv2374sup1.cif

Structure factors: contains datablocks I. DOI: 10.1107/S160053681100242X/pv2374Isup2.hkl

Additional supplementary materials:  crystallographic information; 3D view; checkCIF report

## Figures and Tables

**Table 1:** Hydrogen-bond geometry (Å, °)

*D*—H⋯*A*	*D*—H	H⋯*A*	*D*⋯*A*	*D*—H⋯*A*
O1—H1*B*⋯N2	0.82	1.89	2.705 (2)	171
O4—H4*A*⋯O3	0.82	2.22	2.897 (2)	141
N1—H1*A*⋯O4	0.93 (3)	1.94	2.778 (3)	155
O5—H5*B*⋯O1	0.95 (5)	2.01	2.874 (2)	165
O5—H5*C*⋯O2	0.84 (4)	1.97	2.844 (3)	179

**Fig. 1.**
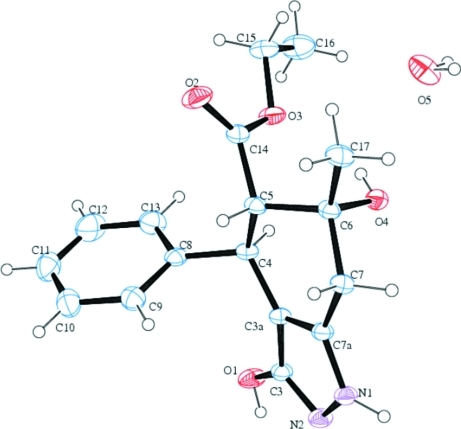
The molecular structure of the title compound, with the atomic numbering scheme. Displacement ellipsoids were drawn at the 30% probability level.

**Fig. 2.**
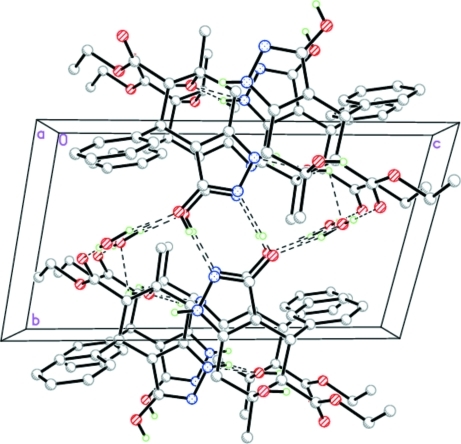
The hydrogen-bonded (dashed lines) packing in the title compound; H-atoms not involved in hydrogen bonding have been excluded for clarity.

## supplementary crystallographic information

### Comment

In the field of heterocyclic compounds, azoles are important due to their wide range of applications (Genin *et al.*, 2000). In the microbial evaluation of organic compounds for the development of current research in drug discovery and medicinal chemistry we have prepared the title compound and determined its crystal structure which has been presented in this article.

In the title compound (Fig. 1), the cyclohexene ring adopts a half-chair conformation, C6 lies 0.685 (3) Å out of the plane formed by the rest of the ring atoms.  The indazole ring (N1/N2/C3/C3A/C7A) is essentially planar with maximum deviation from the ring plane being 0.0192 (12) Å for C7A. In the crystal structure, intermolecular hydrogen bonds O1—H1B···N2 result in centrosymmetric dimers lying about inversion centers. Intramolecular hydrogen bonds O4—H4A···O3  result in six-membered rings. The dimers are further packed and stabilized by N—H···O and O—H···O hydrogen bonds (Table 1 and Fig. 2).

The molecular dimensions in the title compound are in close agreement with the corresponding molecular dimensions of a closely related compoud (Hema *et al.*, 2006).

### Experimental

(rac)-Diethyl-4-hydroxy-4-methyl-6-oxo-2-phenyl-1,3-dicarboxylate (20 mmol) and hydroxylamine hydrochloride (20 mmol) were dissolved in 20 ml e thanol. The mixture was stirred at 345–350 K for 10 h. After cooling to a room temperature colorless crystals were obtained which were filtered and washed with ethanol. The crystals were dissolved in ethanol (50 ml) and recrystallized to yield colourless block-shaped crystals of the title compound suitable for X-ray crystallographic analysis..

### Refinement

The hydrogen atoms of the water of hydration and amino group were localized from difference-Fourier maps and included in the refinement with isotropic displacement parameters. The rest of the hydrogen atoms were placed in calculated positions with and refined in the riding model at O—H = 0.82 and C—H = 0.93-0.98 Å with isotropic displacement parameters *U*_iso_(H) = 1.2 or 1.5*U*_eq_(parent atoms).

### Crystal data

**Table:**

C_17_H_20_N_2_O_4_·H_2_O	*Z* = 2
*M**_r_* = 334.37	*F*(000) = 356
Triclinic, *P*1	*D*_x_ = 1.315 Mg m^−^^3^
Hall symbol: -P 1	Mo *K*α radiation, λ = 0.71073 Å
*a* = 6.9964 (15) Å	Cell parameters from 909 reflections
*b* = 8.8647 (19) Å	θ = 2.5–30.6°
*c* = 15.124 (4) Å	µ = 0.10 mm^−^^1^
α = 99.363 (6)°	*T* = 296 K
β = 95.281 (6)°	Prism, colourless
γ = 112.271 (4)°	0.20 × 0.20 × 0.20 mm
*V* = 844.2 (3) Å^3^	

### Data collection

**Table:**

Bruker APEXII CCD diffractometer	2889 independent reflections
Radiation source: fine-focus sealed tube	2327 reflections with *I* > 2σ(*I*)
graphite	*R*_int_ = 0.065
φ and ω scans	θ_max_ = 25.0°, θ_min_ = 2.5°
Absorption correction: multi-scan (*SADABS*; Bruker, 2003)	*h* = −8→8
*T*_min_ = 0.981, *T*_max_ = 0.981	*k* = −10→10
6332 measured reflections	*l* = −17→16

### Refinement

**Table:**

Refinement on *F*^2^	Primary atom site location: structure-invariant direct methods
Least-squares matrix: full	Secondary atom site location: difference Fourier map
*R*[*F*^2^ > 2σ(*F*^2^)] = 0.077	Hydrogen site location: difference Fourier map
*wR*(*F*^2^) = 0.198	H atoms treated by a mixture of independent and constrained refinement
*S* = 1.00	*w* = 1/[σ^2^(*F*_o_^2^) + (0.0611*P*)^2^ + 0.4352*P*] where *P* = (*F*_o_^2^ + 2*F*_c_^2^)/3
2889 reflections	(Δ/σ)_max_ < 0.001
231 parameters	Δρ_max_ = 0.27 e Å^−^^3^
0 restraints	Δρ_min_ = −0.37 e Å^−^^3^

### Special details

**Table:**

Geometry. All e.s.d.'s (except the e.s.d. in the dihedral angle between two l.s. planes) are estimated using the full covariance matrix. The cell e.s.d.'s are taken into account individually in the estimation of e.s.d.'s in distances, angles and torsion angles; correlations between e.s.d.'s in cell parameters are only used when they are defined by crystal symmetry. An approximate (isotropic) treatment of cell e.s.d.'s is used for estimating e.s.d.'s involving l.s. planes.
Refinement. Refinement of *F*^2^ against ALL reflections. The weighted *R*-factor *wR* and goodness of fit *S* are based on *F*^2^, conventional *R*-factors *R* are based on *F*, with *F* set to zero for negative *F*^2^. The threshold expression of *F*^2^ > σ(*F*^2^) is used only for calculating *R*-factors(gt) *etc*. and is not relevant to the choice of reflections for refinement. *R*-factors based on *F*^2^ are statistically about twice as large as those based on *F*, and *R*- factors based on ALL data will be even larger.

### Fractional atomic coordinates and isotropic or equivalent isotropic displacement parameters (Å^2^)

**Table:**

	*x*	*y*	*z*	*U*_iso_*/*U*_eq_	
O1	0.9685 (3)	1.3998 (2)	0.37394 (14)	0.0463 (5)	
H1B	0.9998	1.4897	0.4089	0.069*	
O2	0.7109 (3)	0.6179 (2)	0.15151 (15)	0.0544 (6)	
O3	0.4935 (3)	0.7477 (2)	0.14842 (13)	0.0421 (5)	
O4	0.3968 (2)	0.8009 (2)	0.33029 (13)	0.0363 (5)	
H4A	0.3737	0.7989	0.2759	0.054*	
O5	0.0528 (4)	0.5446 (3)	0.2177 (2)	0.0637 (7)	
H5B	0.009 (6)	0.485 (6)	0.264 (3)	0.081 (13)*	
H5C	−0.051 (6)	0.563 (5)	0.198 (3)	0.068 (11)*	
N1	0.8141 (3)	1.1503 (2)	0.52847 (16)	0.0340 (5)	
N2	0.8884 (3)	1.3042 (2)	0.50553 (16)	0.0373 (6)	
C3	0.8995 (4)	1.2791 (3)	0.41766 (18)	0.0326 (6)	
C3A	0.8266 (3)	1.1038 (3)	0.38069 (17)	0.0302 (6)	
C4	0.7975 (3)	1.0105 (3)	0.28485 (17)	0.0306 (6)	
H4B	0.6826	1.0230	0.2490	0.037*	
C5	0.7311 (3)	0.8213 (3)	0.28392 (18)	0.0312 (6)	
H5A	0.8575	0.8062	0.3058	0.037*	
C6	0.5710 (3)	0.7581 (3)	0.34881 (18)	0.0316 (6)	
C7	0.6747 (4)	0.8481 (3)	0.44704 (18)	0.0325 (6)	
H7A	0.5710	0.8228	0.4869	0.039*	
H7B	0.7819	0.8107	0.4667	0.039*	
C7A	0.7705 (3)	1.0317 (3)	0.45193 (17)	0.0307 (6)	
C8	0.9887 (4)	1.0726 (3)	0.23964 (18)	0.0327 (6)	
C9	1.1870 (4)	1.1056 (3)	0.2856 (2)	0.0430 (7)	
H9A	1.2015	1.0902	0.3449	0.052*	
C10	1.3610 (5)	1.1603 (3)	0.2451 (3)	0.0546 (9)	
H10A	1.4921	1.1823	0.2771	0.066*	
C11	1.3422 (5)	1.1827 (4)	0.1572 (3)	0.0635 (10)	
H11A	1.4600	1.2185	0.1294	0.076*	
C12	1.1468 (6)	1.1516 (4)	0.1104 (3)	0.0690 (10)	
H12A	1.1332	1.1688	0.0515	0.083*	
C13	0.9724 (5)	1.0950 (4)	0.1516 (2)	0.0519 (8)	
H13A	0.8412	1.0715	0.1193	0.062*	
C14	0.6486 (4)	0.7183 (3)	0.18762 (19)	0.0350 (6)	
C15	0.3862 (5)	0.6480 (4)	0.0578 (2)	0.0511 (8)	
H15A	0.3097	0.5325	0.0606	0.061*	
H15B	0.4869	0.6527	0.0172	0.061*	
C16	0.2398 (6)	0.7190 (5)	0.0244 (3)	0.0693 (10)	
H16A	0.1663	0.6560	−0.0352	0.104*	
H16B	0.3173	0.8331	0.0217	0.104*	
H16C	0.1409	0.7137	0.0650	0.104*	
C17	0.4995 (4)	0.5701 (3)	0.3400 (2)	0.0432 (7)	
H17A	0.4114	0.5349	0.3842	0.065*	
H17B	0.6197	0.5438	0.3502	0.065*	
H17C	0.4224	0.5133	0.2800	0.065*	
H1A	0.760 (4)	1.143 (3)	0.582 (2)	0.034 (7)*	

### Atomic displacement parameters (Å^2^)

**Table:**

	*U*^11^	*U*^22^	*U*^33^	*U*^12^	*U*^13^	*U*^23^
O1	0.0653 (12)	0.0205 (8)	0.0410 (13)	0.0057 (8)	0.0101 (9)	0.0015 (8)
O2	0.0622 (12)	0.0460 (11)	0.0529 (15)	0.0319 (10)	−0.0016 (10)	−0.0141 (10)
O3	0.0449 (10)	0.0372 (9)	0.0360 (12)	0.0172 (8)	−0.0059 (8)	−0.0085 (8)
O4	0.0331 (9)	0.0360 (9)	0.0361 (11)	0.0138 (7)	0.0014 (7)	0.0008 (8)
O5	0.0449 (12)	0.0594 (14)	0.075 (2)	0.0095 (10)	−0.0053 (12)	0.0196 (13)
N1	0.0382 (11)	0.0261 (10)	0.0290 (14)	0.0056 (8)	0.0054 (9)	0.0006 (9)
N2	0.0442 (12)	0.0233 (10)	0.0366 (15)	0.0075 (8)	0.0055 (9)	0.0015 (9)
C3	0.0356 (12)	0.0247 (11)	0.0302 (15)	0.0065 (9)	0.0043 (10)	0.0011 (10)
C3A	0.0316 (11)	0.0210 (11)	0.0312 (15)	0.0065 (8)	0.0028 (9)	−0.0015 (10)
C4	0.0349 (12)	0.0222 (11)	0.0304 (15)	0.0095 (9)	0.0001 (10)	0.0020 (9)
C5	0.0313 (11)	0.0212 (11)	0.0361 (16)	0.0089 (9)	0.0009 (10)	−0.0002 (10)
C6	0.0315 (11)	0.0227 (11)	0.0352 (16)	0.0079 (9)	0.0020 (10)	0.0012 (10)
C7	0.0339 (12)	0.0258 (11)	0.0337 (16)	0.0085 (9)	0.0037 (10)	0.0053 (10)
C7A	0.0291 (11)	0.0249 (11)	0.0316 (15)	0.0082 (9)	−0.0002 (9)	−0.0020 (10)
C8	0.0404 (13)	0.0190 (10)	0.0341 (16)	0.0097 (9)	0.0057 (10)	−0.0003 (10)
C9	0.0459 (15)	0.0370 (13)	0.0455 (19)	0.0168 (11)	0.0067 (12)	0.0072 (12)
C10	0.0438 (15)	0.0390 (15)	0.081 (3)	0.0164 (12)	0.0190 (15)	0.0091 (15)
C11	0.063 (2)	0.0436 (16)	0.080 (3)	0.0124 (14)	0.0382 (19)	0.0118 (17)
C12	0.087 (3)	0.062 (2)	0.048 (2)	0.0133 (18)	0.0266 (18)	0.0183 (17)
C13	0.0565 (17)	0.0467 (16)	0.044 (2)	0.0117 (13)	0.0081 (14)	0.0102 (14)
C14	0.0347 (12)	0.0235 (11)	0.0412 (17)	0.0080 (9)	0.0060 (11)	0.0015 (10)
C15	0.0574 (17)	0.0436 (15)	0.0371 (19)	0.0143 (13)	−0.0075 (13)	−0.0090 (13)
C16	0.074 (2)	0.070 (2)	0.053 (2)	0.0300 (18)	−0.0157 (17)	−0.0018 (17)
C17	0.0453 (14)	0.0245 (12)	0.051 (2)	0.0060 (10)	0.0055 (12)	0.0053 (11)

### Geometric parameters (Å, °)

**Table:**

O1—C3	1.308 (3)	C7—C7A	1.493 (3)
O1—H1B	0.8200	C7—H7A	0.9700
O2—C14	1.209 (3)	C7—H7B	0.9700
O3—C14	1.321 (3)	C8—C13	1.379 (4)
O3—C15	1.462 (3)	C8—C9	1.394 (4)
O4—C6	1.425 (3)	C9—C10	1.371 (4)
O4—H4A	0.8200	C9—H9A	0.9300
O5—H5B	0.95 (5)	C10—C11	1.378 (5)
O5—H5C	0.85 (4)	C10—H10A	0.9300
N1—C7A	1.354 (3)	C11—C12	1.385 (6)
N1—N2	1.379 (3)	C11—H11A	0.9300
N1—H1A	0.92 (3)	C12—C13	1.381 (5)
N2—C3	1.326 (4)	C12—H12A	0.9300
C3—C3A	1.432 (3)	C13—H13A	0.9300
C3A—C7A	1.355 (4)	C15—C16	1.482 (5)
C3A—C4	1.502 (3)	C15—H15A	0.9700
C4—C8	1.514 (3)	C15—H15B	0.9700
C4—C5	1.558 (3)	C16—H16A	0.9600
C4—H4B	0.9800	C16—H16B	0.9600
C5—C14	1.517 (3)	C16—H16C	0.9600
C5—C6	1.561 (4)	C17—H17A	0.9600
C5—H5A	0.9800	C17—H17B	0.9600
C6—C17	1.526 (3)	C17—H17C	0.9600
C6—C7	1.532 (3)		
			
C3—O1—H1B	109.5	C3A—C7A—C7	125.0 (2)
C14—O3—C15	117.9 (2)	C13—C8—C9	117.9 (3)
C6—O4—H4A	109.5	C13—C8—C4	121.4 (2)
H5B—O5—H5C	105 (4)	C9—C8—C4	120.6 (2)
C7A—N1—N2	108.2 (2)	C10—C9—C8	121.2 (3)
C7A—N1—H1A	130.0 (17)	C10—C9—H9A	119.4
N2—N1—H1A	117.5 (17)	C8—C9—H9A	119.4
C3—N2—N1	107.5 (2)	C9—C10—C11	120.2 (3)
O1—C3—N2	123.5 (2)	C9—C10—H10A	119.9
O1—C3—C3A	126.8 (2)	C11—C10—H10A	119.9
N2—C3—C3A	109.6 (2)	C10—C11—C12	119.6 (3)
C7A—C3A—C3	104.3 (2)	C10—C11—H11A	120.2
C7A—C3A—C4	124.9 (2)	C12—C11—H11A	120.2
C3—C3A—C4	130.6 (2)	C13—C12—C11	119.6 (4)
C3A—C4—C8	114.11 (18)	C13—C12—H12A	120.2
C3A—C4—C5	109.1 (2)	C11—C12—H12A	120.2
C8—C4—C5	109.85 (19)	C8—C13—C12	121.4 (3)
C3A—C4—H4B	107.8	C8—C13—H13A	119.3
C8—C4—H4B	107.8	C12—C13—H13A	119.3
C5—C4—H4B	107.8	O2—C14—O3	123.6 (2)
C14—C5—C4	110.5 (2)	O2—C14—C5	125.0 (2)
C14—C5—C6	111.47 (18)	O3—C14—C5	111.4 (2)
C4—C5—C6	112.80 (19)	O3—C15—C16	107.3 (2)
C14—C5—H5A	107.2	O3—C15—H15A	110.3
C4—C5—H5A	107.2	C16—C15—H15A	110.3
C6—C5—H5A	107.2	O3—C15—H15B	110.3
O4—C6—C17	111.01 (18)	C16—C15—H15B	110.3
O4—C6—C7	106.04 (18)	H15A—C15—H15B	108.5
C17—C6—C7	109.6 (2)	C15—C16—H16A	109.5
O4—C6—C5	110.4 (2)	C15—C16—H16B	109.5
C17—C6—C5	110.5 (2)	H16A—C16—H16B	109.5
C7—C6—C5	109.18 (18)	C15—C16—H16C	109.5
C7A—C7—C6	109.0 (2)	H16A—C16—H16C	109.5
C7A—C7—H7A	109.9	H16B—C16—H16C	109.5
C6—C7—H7A	109.9	C6—C17—H17A	109.5
C7A—C7—H7B	109.9	C6—C17—H17B	109.5
C6—C7—H7B	109.9	H17A—C17—H17B	109.5
H7A—C7—H7B	108.3	C6—C17—H17C	109.5
N1—C7A—C3A	110.2 (2)	H17A—C17—H17C	109.5
N1—C7A—C7	124.8 (2)	H17B—C17—H17C	109.5
			
C7A—N1—N2—C3	−3.1 (3)	C3—C3A—C7A—N1	−3.0 (3)
N1—N2—C3—O1	−178.5 (2)	C4—C3A—C7A—N1	−178.8 (2)
N1—N2—C3—C3A	1.2 (3)	C3—C3A—C7A—C7	177.1 (2)
O1—C3—C3A—C7A	−179.2 (2)	C4—C3A—C7A—C7	1.3 (4)
N2—C3—C3A—C7A	1.1 (3)	C6—C7—C7A—N1	157.6 (2)
O1—C3—C3A—C4	−3.8 (4)	C6—C7—C7A—C3A	−22.5 (3)
N2—C3—C3A—C4	176.5 (2)	C3A—C4—C8—C13	−135.7 (2)
C7A—C3A—C4—C8	−133.3 (2)	C5—C4—C8—C13	101.3 (3)
C3—C3A—C4—C8	52.1 (3)	C3A—C4—C8—C9	45.6 (3)
C7A—C3A—C4—C5	−10.0 (3)	C5—C4—C8—C9	−77.3 (3)
C3—C3A—C4—C5	175.4 (2)	C13—C8—C9—C10	0.7 (4)
C3A—C4—C5—C14	165.98 (18)	C4—C8—C9—C10	179.3 (2)
C8—C4—C5—C14	−68.2 (2)	C8—C9—C10—C11	−0.4 (4)
C3A—C4—C5—C6	40.4 (2)	C9—C10—C11—C12	0.8 (5)
C8—C4—C5—C6	166.2 (2)	C10—C11—C12—C13	−1.4 (5)
C14—C5—C6—O4	−72.4 (2)	C9—C8—C13—C12	−1.3 (4)
C4—C5—C6—O4	52.7 (2)	C4—C8—C13—C12	−180.0 (3)
C14—C5—C6—C17	50.8 (3)	C11—C12—C13—C8	1.7 (5)
C4—C5—C6—C17	175.87 (19)	C15—O3—C14—O2	2.6 (4)
C14—C5—C6—C7	171.44 (19)	C15—O3—C14—C5	−175.3 (2)
C4—C5—C6—C7	−63.5 (2)	C4—C5—C14—O2	126.8 (3)
O4—C6—C7—C7A	−68.0 (2)	C6—C5—C14—O2	−106.9 (3)
C17—C6—C7—C7A	172.1 (2)	C4—C5—C14—O3	−55.4 (3)
C5—C6—C7—C7A	50.9 (2)	C6—C5—C14—O3	70.9 (3)
N2—N1—C7A—C3A	3.9 (3)	C14—O3—C15—C16	−172.8 (3)
N2—N1—C7A—C7	−176.2 (2)		

### Hydrogen-bond geometry (Å, °)

**Table:**

*D*—H···*A*	*D*—H	H···*A*	*D*···*A*	*D*—H···*A*
O1—H1B···N2^i^	0.82	1.89	2.705 (2)	171
O4—H4A···O3^i^	0.82	2.22	2.897 (2)	141
N1—H1A···O4^ii^	0.93 (3)	1.94	2.778 (3)	155
O5—H5B···O1^i^	0.95 (5)	2.01	2.874 (2)	165
O5—H5C···O2^ii^	0.84 (4)	1.97	2.844 (3)	179

Symmetry codes: (i) ; (ii) .
